# MGnify Genomes: A Resource for Biome-specific Microbial Genome Catalogues

**DOI:** 10.1016/j.jmb.2023.168016

**Published:** 2023-07-15

**Authors:** Tatiana A. Gurbich, Alexandre Almeida, Martin Beracochea, Tony Burdett, Josephine Burgin, Guy Cochrane, Shriya Raj, Lorna Richardson, Alexander B. Rogers, Ekaterina Sakharova, Gustavo A. Salazar, Robert D. Finn

**Affiliations:** 1 European Molecular Biology Laboratory, European Bioinformatics Institute (EMBL-EBI), Wellcome Genome Campus, Hinxton, UK; 2 Department of Veterinary Medicine, University of Cambridge, Cambridge, UK

**Keywords:** Microbiome, Functional annotation, Metagenome-assembled genome, Uncultured microorganisms, Biodiversity

## Abstract

An increasingly common output arising from the analysis of shotgun metagenomic datasets is the generation of metagenome-assembled genomes (MAGs), with tens of thousands of MAGs now described in the literature. However, the discovery and comparison of these MAG collections is hampered by the lack of uniformity in their generation, annotation and storage. To address this, we have developed MGnify Genomes, a growing collection of biome-specific non-redundant microbial genome catalogues generated using MAGs and publicly available isolate genomes.

Genomes within a biome-specific catalogue are organised into species clusters. For species that contain multiple conspecific genomes, the highest quality genome is selected as the representative, always prioritising an isolate genome over a MAG. The species representative sequences and annotations can be visualised on the MGnify website and the full catalogue and associated analysis outputs can be downloaded from MGnify servers. A suite of online search tools is provided allowing users to compare their own sequences, ranging from a gene to sets of genomes, against the catalogues.

Seven biomes are available currently, comprising over 300,000 genomes that represent 11,048 non-redundant species, and include 36 taxonomic classes not currently represented by cultured genomes. MGnify Genomes is available at https://www.ebi.ac.uk/metagenomics/browse/genomes/.

## Highlights

• MGnify Genomes is a free-to-use resource for biome-specific microbial genomes.
• Each genome catalogue is non-redundant and includes extensive functional annotations.
• Users can search their own sequences against the resource to assess their novelty.
• MGnify Genomes can be accessed via the website, the FTP server and the API.
• The resource provides insight into previously uncultured species and novel proteins.

## Graphical abstract

**Figure:**
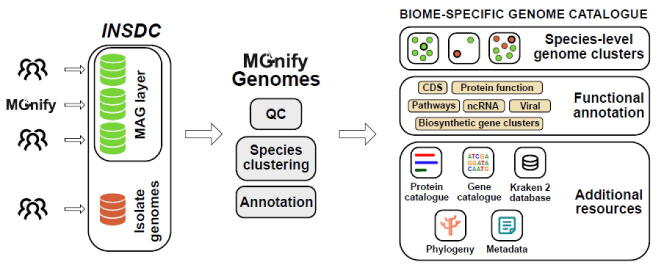

## Introduction

The recent years have witnessed a paradigm shift in metagenomic research as a consequence of increased sequencing depth concomitant with the development and improvement of tools for metagenomic assembly and binning, moving the focus towards genome-resolved analysis.^1^ Large-scale projects, such as *Tara* Oceans and Earth’s Microbiomes, have produced several thousand metagenome-assembled genomes (MAGs) as part of their efforts to characterise microbial diversity across marine and terrestrial biomes respectively.^2^, ^3^, ^4^ Several other MAG catalogues exist for host-associated biomes, including the human gut,^5^, ^6^ human skin^7^ and animal-associated microbiomes.^8^, ^9^ Despite the best efforts of the European Nucleotide Archive (ENA) to provide specific MAG submission layers for such MAG collections, many community MAG catalogues are not yet routinely submitted in ENA, often being made available via general purpose repositories such as Zenodo or Figshare, which limits their discovery. Furthermore, even if they are submitted to ENA, there is inevitable variation across collections in terms of quality checking, redundancy removal and application of taxonomic classification. Finally, few, if any, collections contain functional annotations associated with the MAGs. Consequently, the majority of this newly identified biodiversity is not easily accessible for contextualising similar datasets, frustrating efforts to determine genomes that may be common or unique between samples. To overcome these obstacles, we have extended the MGnify resource to create a dedicated hub for cataloguing microbial genomes found in particular biomes, facilitating easy access to explore and download quality-filtered genomes and corresponding functional annotations.

In addition to ENA, several other resources exist where users can explore biome-specific data or submit their own. For example, the Integrated Microbial Genomes and Microbiomes (IMG/M) data management system^10^ is an expansive database that features both isolate and binned metagenomes along with other types of microbiome data and allows user data submission. The bins are annotated with the biome from which they are derived and can be browsed as biome-specific catalogues, however, these catalogues are not dereplicated and have limited coverage of human-associated biomes due to their scientific focus. Similarly, the proGenomes resource facilitates the exploration of biome-specific microbial genomes and their functional annotations, however, the resource is currently focused on isolate genomes and does not include MAGs as yet.^11^ The Microbial Genomes Atlas (MiGA) provides data and tools to classify and catalogue genomes (including MAGs) against selected publicly available MAGs and reference genomes.^12^ Finally, there are some examples of single-biome resources that serve a more targeted audience, for example, MarRef and MarDB which capture genomes from marine associated resources.^13^

Here we describe MGnify Genomes, a free to use resource which contains biome-specific non-redundant quality-controlled microbial genome catalogues generated from publicly available data. MGnify Genomes is an extension to the MGnify platform,^14^ which provides the assembly, analysis, and archiving of microbiome-derived sequences. Development of MGnify Genomes is combined with updates to the existing catalogues as well as the addition of further biome-specific catalogues as appropriate datasets become available. Herein, we describe the construction and content of the initial set of genome catalogues.

## Results

MGnify Genomes catalogues are primarily composed of MAGs, both user-submitted and those generated by MGnify, which have been deposited in ENA. Where an appropriate curated collection of biome-specific isolate genomes exists, the catalogues may also contain isolate genomes to enhance the diversity and completeness of the catalogue. Our aim is to capture higher genomic quality MAGs, and since ENA stipulates that submitted MAGs should contain less than 10% contamination with no constraints on completeness, we filter MAGs according to the QS50 criteria described below, using the user-defined completeness and contamination estimates included in the submission metadata.

All MAGs within the catalogues are of at least medium quality as per the MIMAG standards^15^ with additional quality filters to limit contamination to a maximum of 5% and the overall quality score of each genome, calculated as % completeness – 5 × % contamination, to at least 50. Genomes within each catalogue are organised into species-level clusters based on an average nucleotide identity of 95% with 30% minimum overlap between genomes, such that genomes in each cluster represent strains of the same species. Within each cluster, the highest quality genome is designated as the species representative (SR), always prioritising an isolate genome over a MAG irrespective of genome quality estimates. These genome sets are processed using a standardised versioned pipeline providing taxonomic, functional and pan-genome annotations, as well as associated sequence files (described below).

### Current catalogues

**Figure 1:**
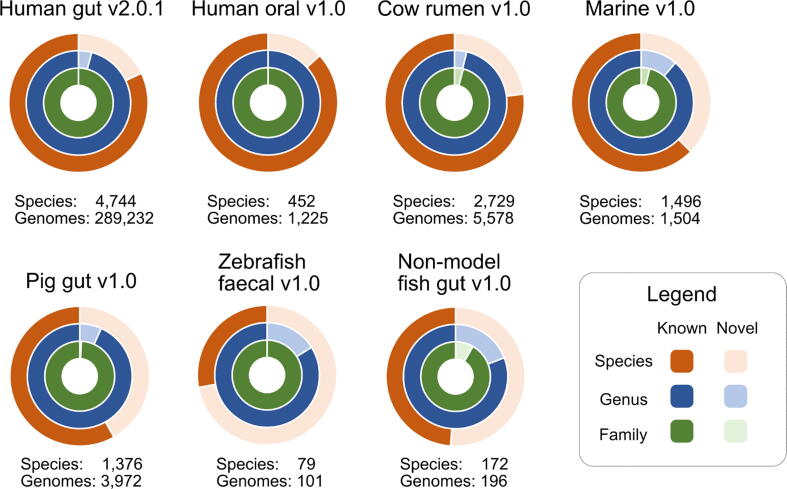
Summary of the current catalogues available in MGnify Genomes, their sizes and degree of novelty therein. Novelty at species, genus and family level with respect to GTDB r207 (which includes isolates and MAGs, some of which have come from these catalogues and have been taxonomically labelled by GTDB) is shown for each catalogue, with novelty defined here as the absence of a named taxon in GTDB. When the genus or family levels are unknown, determining whether two novel genomes belong to the same novel taxon or different ones is not always possible, which could inflate the number of novel genera and families.

Seven catalogues are currently available in MGnify. In total, they contain 301,808 genomes and 11,048 SRs, the latter of which are presented and searchable via the website, while all data are available via the FTP site. The human gut catalogue^6^ is currently the largest with 289,232 genomes and 4,744 SRs. The complete breakdown of each catalogue is illustrated in Figure 1.

Of the seven catalogues, the human gut and the cow rumen^16^ are based on existing published catalogue resources, with the latter comprising solely of third-party-generated MAGs. The remaining catalogues were generated using multiple studies obtained from ENA and NCBI’s GenBank.^17^ The README file for each catalogue includes a list of the International Nucleotide Sequence Database Collaboration (INSDC) study accessions from which the genomes were obtained. When choosing biomes for the initial set of catalogues, we focused on those that already have large collections of metagenomic data available (e.g. different human microbiomes and the pig gut) as well as biomes that are studied as part of collaborative projects that MGnify is involved in (e.g. marine and fish gut). The catalogues are versioned; notably the human gut catalogue has been updated with new studies since it first became available (see methods for the update process description), thereby allowing users to clearly reference the dataset used to enhance provenances. The latest version of each catalogue is always presented on the website, with older versions available on the FTP site. Minor point updates in versioning reflect additional files or minor bug fixes.

### Catalogue organisation

Each genome within a catalogue is represented by a general feature format (GFF) file containing the genomic sequence, predicted coding sequences (CDS) and associated functional annotations generated by Prokka.^18^ These GFF files are available from the MGnify FTP server, which is linked from the catalogue webpage, accessed via https://www.ebi.ac.uk/metagenomics/browse/genomes.

SR genomes have additional functional annotations provided. CDS are annotated with InterPro,^19^ eggNOG,^20^ Pfam,^21^ COG category,^22^ KEGG orthology,^23^ and CAZy^24^ to provide insight into protein function. Non-coding RNAs are identified and annotated using Infernal^25^ and Rfam^26^ and, where present, rRNA sequences are provided in FASTA format. The zebrafish faecal, non-model fish gut and pig gut catalogues, which are generated using MGnify Genomes pipeline v1.3.1, also include biosynthetic gene cluster predictions and viral annotations (see methods). Annotated GFF files relating to SRs can be viewed using the IGV genome browser^27^ on the MGnify Genomes website, as well as downloaded (alongside all other files) from the catalogue-specific folder on the MGnify FTP server. For species clusters that consist of multiple conspecific genomes, we provide a pan-genome with all genes from all conspecific genomes as a FASTA file, along with a list of core genes and a table listing the presence of each gene per genome in the cluster.

Each biome-specific catalogue has a corresponding non-redundant protein catalogue with the protein sequences clustered at 50%, 90%, 95%, and 100% amino acid similarity. InterPro and eggNOG annotations are provided for the 90% identity clustering. Catalogues generated using pipeline v1.3.1 also include a gene catalogue which contains the nucleotide sequence for each protein cluster representative of the 100% identity clustering. These resources provide further ways to functionally characterise the organisms within catalogues and facilitate analysis on specific genes or proteins.

We provide a Mash sketch^28^ generated from all genomes within a catalogue, allowing users to compare their own sequences against the catalogue sketches using Mash. We also provide a Kraken 2^29^ and a Bracken^30^ database with each catalogue enabling users to classify DNA sequence reads or contigs using the catalogue taxonomy. A phylogenetic tree of SRs from each catalogue can be visualised using the IQ-TREE 2^31^ file provided, and we also include GTDB-Tk-generated^32^ multiple-sequence alignments of SRs allowing users to generate their own trees. A GTDB-based taxonomy tree for each catalogue is available on the website enabling users to locate genomes from taxa of interest.

Lastly, each catalogue comes with an extensive metadata table. For each genome this includes assembly quality information, the species cluster to which the genome belongs, taxonomy, metadata for the sample from which the genome was derived, the number of detected RNA sequences, and a URL from where the GFF file containing the annotation and the genome sequence can be downloaded.

### Searching the catalogues

**Figure 2:**
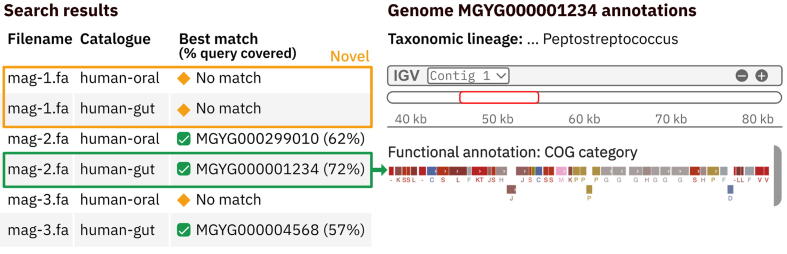
Search results (left) comparing three query genome sequences against two catalogues: human oral and gut. The sourmash search results show that one query genome is novel with respect to the catalogues; one is found in both catalogues; and one is found in only the gut catalogue. Users can access the taxonomic and functional annotations for matching genomes (right).

The MGnify website and API provide two different sequence search functionalities for users to compare either (i) short sequences (i.e. gene) or (ii) individual genomes or collections of genomes against one or more MGnify catalogues (Figure 2). These different search strategies balance search performance (speed) and sensitivity according to the length of the query sequence. The short sequence (50–5,000 bp) search (based on COBS^33^) results include a list of matched genomes and the catalogue in which each can be found, as well as the percentage of matched k-mers between the query and the target. The user can adjust the minimum proportion of query k-mers to be matched to control the level of similarity of matches that are reported.

The genome(s) vs genomes search results (based on sourmash^34^) list the best matching MGnify genome from each target catalogue for every query genome. A report file is also produced listing other secondary matches to allow for further programmatic analysis of similar sequences.

### Catalogue novelty

The culture-independent approach of metagenomics allows a better representation of the microbial community found in an environment compared to what can be achieved with isolation, cultivation and sequencing of genomes alone. To demonstrate the novel genomic diversity being captured in the MGnify Genomes catalogues, we performed a comparison against the isolate genomes in GTDB (see methods).

GTDB draws genomes from RefSeq^35^ and GenBank and includes draft genomes and uncultured organisms.^36^ While some publicly available MAGs incorporated into MGnify Genomes have been well described in associated publications, they can still be novel with respect to GTDB. Therefore, when using GTDB to determine the taxonomic placement of a genome, if the genome cannot be classified to the species level, we define that as an indication of its novelty for the purposes of this publication. By this metric, all catalogues include novel species, ranging from ∼13% in the human oral catalogue to ∼72% in the zebrafish faecal catalogue, with most catalogues also containing novelty at genus and family level (Figure 1). Since GTDB encapsulates GenBank MAGs including those that are part of the MGnify catalogues, more recently generated MAGs that are not yet part of a GTDB release are more likely to be from an unknown taxon.

**Figure 3:**
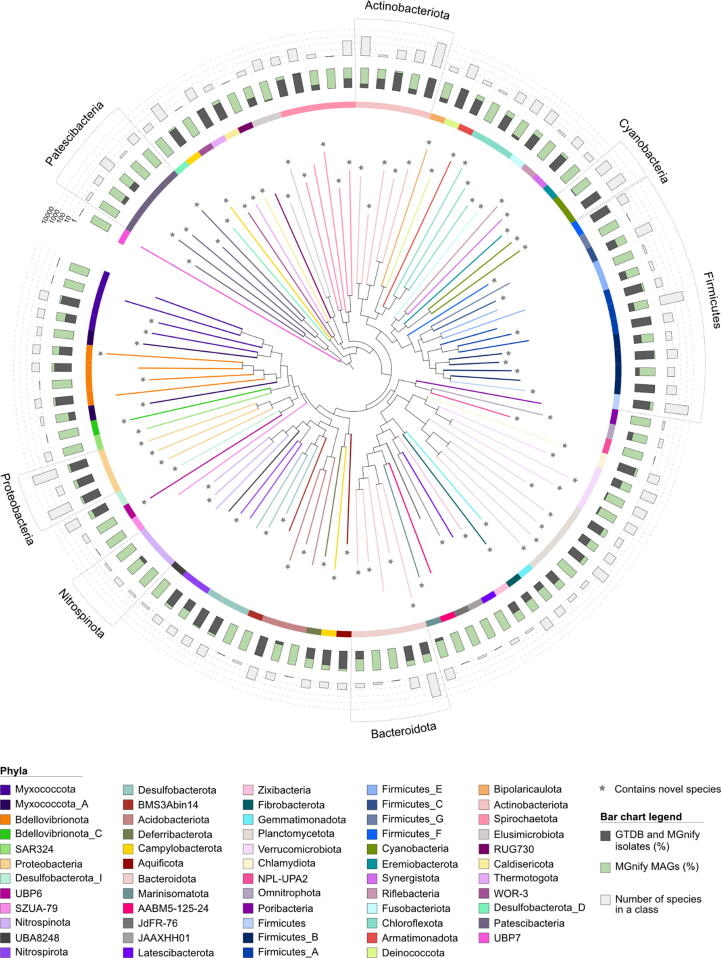
A taxonomic tree showing diversity and novelty introduced to the known bacterial phylogeny by genome catalogues. The tree was generated using bacterial SR isolate genomes from GTDB v207 and MGnify SRs from the seven available catalogues. The highest quality genome per class was chosen to build the tree. Only classes represented in MGnify catalogues are shown. The stacked bar charts show the fraction of genomes in each class that have an isolate SR in MGnify or GTDB and the fraction of genomes represented only by MAGs. The outermost set of bar charts shows the number of species in each class. 36 bacterial classes represented in the catalogues do not yet include species with an isolate representative in GTDB. Stars denote 83 classes that contain MGnify species that are not named in GTDB r207.

To understand novelty from a broader perspective, we evaluated the increased taxonomic diversity captured by the genome catalogues (Figure 3). A representative taxonomic tree was constructed using GTDB isolate genomes and MGnify catalogues (see methods) to depict all 108 taxonomic classes containing MGnify Genomes from the seven catalogues described in this article. Of the 108 classes shown, 36 classes are only represented by MAGs. Furthermore, 83 classes contain at least one uncultured species introduced by MGnify Genomes that does not match any existing genomes in GTDB. Species counts of each of the 108 classes, as well as the breakdown of these into the number of MAGs and isolate genomes in each class are shown in Supplementary Table 1. The relative fractions of MAGs and isolates vary across the taxonomic tree (see Figure 3). Some phyla, e.g. Nitrospinota and Patescibacteria, contain multiple classes that are either uncultured or have a large proportion of novel species per class contributed by MGnify MAGs. Notably there are some large classes contained within specific phyla such as Cyanobacteria (which includes 153 species of Vampirovibrionia and 524 species of Cyanobacteriia) and Actinobacteriota (which includes 798 species of Coriobacteriia and 4,806 species of Actinomycetia), where one class is predominantly represented by cultured species while another is primarily composed of MAGs. Finally, the Bacteroidia class within the Bacteroidota phylum is already one of the most abundant classes in the human gut with thousands of known strains.^6^ Unsurprisingly, it is also well represented in this taxonomic tree with 4,167 species of which 1,594 are MAGs that do not have an isolate SR in GTDB. Overall, this not only highlights the broad taxonomic distribution of the MGnify Genomes, but also indicates areas of potential future research to understand these microbes better and taxonomic classes of microbes that would benefit from targeted cultivation efforts.

## Discussion

As metagenomic methods continue to improve, from the sequencing technology employed to the informatics analysis, the number of MAGs produced by the scientific community and their quality is anticipated to increase. To understand microbial biodiversity in a comprehensive fashion, it is essential to facilitate the availability of high-quality systematic and consistent reference genome datasets for comparative purposes. MGnify Genomes represents the first steps towards establishing such a comprehensive and reliable resource. The MGnify team will not only continue to produce MAGs for incorporation into both existing and new catalogues representing more diverse environments such as fresh water, but is already collaborating with many producers of MAGs to ensure that their genomes are also represented in MGnify catalogues. Nevertheless, highly diverse environments like soil, which are less tractable to assembly and MAG generation, as well as those with fewer sequenced samples will take longer to be represented in MGnify catalogues. All genomes must be deposited in an INSDC database before they can be incorporated into MGnify, and we strongly encourage the wider scientific community to submit their own MAGs to the archives. As such, we anticipate the number of MGnify genome catalogues to increase along with an expansion in the number of genomes in each catalogue. New genomes will be included periodically, based on when there are notable depositions of new MAGs, requests from the MGnify user community and through funded collaborations. By systematically producing and publishing these catalogues, we not only ensure that metrics such as species diversity are comparable and not a reflection of differing thresholds or approaches to quality control, but also increase their discoverability and accessibility by the scientific community. We continue to monitor, participate in and adopt community efforts to extend taxonomic classifications to include uncultivated organisms, such as those being advocated by GTDB.

We anticipate many different applications for the MGnify catalogues that go beyond comparisons of the taxa and functions contained within two or more biomes. For example, our catalogues offer alternative referenced-based approaches for analysing unassembled metagenomics datasets. While this may not surpass *de novo* assembly, it will provide a computationally efficient way to identify species that are likely to be contained within a dataset, or discover metagenomic datasets with a very different composition. MGnify Genomes will also provide new reference datasets for resources, such as MetaPhlAn^37^ or mOTUs,^38^ as well as support the development of new tools for delineating strains by providing training data. Finally, we will consider new biomes for inclusion in the resource that represent not only our own research interests but also reflect community needs. The MGnify team welcomes any input or collaborations from the community.

## Materials and Methods

### Data

We generate genome catalogues using public metagenomic and isolate genome datasets available in the INSDC initiative. To identify metagenomic samples from a biome of interest, we use study metadata in INSDC and, where available, the accompanying publication.

For metagenomic datasets that do not already contain user-submitted MAGs or metagenomic assemblies, we assemble shotgun metagenome sequencing reads using metaSPAdes,^39^ and identify and remove phiX174 bacteriophage and host-associated contigs (where appropriate) using BLAST.^40^ Subsequently, we bin the contigs using metaWRAP’s^41^ binning module (with --metabat2 --concoct --maxbin2 options and the minimum contig length set to 2,500 base pairs), refine the bins using metaWRAP’s bin_refinement module (with -c 50 -x 5 options, discarding any bins that contain less than 50% genome completeness or contain greater than 5% contamination), and assess final bin quality using CheckM.^42^ Additionally, we identify bins that are likely chimeric using GUNC^43^ and remove bins matching all of the following criteria: clade separation score >0.45, contamination portion >0.05, reference representation score >0.5, <90% complete. We then dereplicate the bins to remove equivalences at the species level using dRep,^44^ setting the average nucleotide identity cutoff to 95% to keep the highest quality MAG at this level of sequence similarity, and setting minimum alignment fraction to 0.6. All quality controlled MAGs produced by MGnify are uploaded to ENA. The tool versions used to generate each MAG are listed in its metadata in ENA.

### Catalogue generation

In an effort to make the catalogues FAIR,^45^ the code for the genome catalogue pipeline is available at https://github.com/EBI-Metagenomics/genomes-pipeline/. The version of the pipeline used to generate each catalogue is specified on the corresponding catalogue webpage. Newer catalogues may be generated with different pipeline versions, as we introduce new features and update reference databases, without having the onerous task of re-running every catalogue. Tool versions used to produce each catalogue are listed in the associated README file on the FTP server.

To generate a genome catalogue, we collect all user-submitted and MGnify-produced MAGs (and where possible isolate genomes) from a biome of interest and filter them using CheckM or, where available, user-submitted completeness and contamination information, to remove genomes that are less than 50% complete, more than 5% contaminated or have QS score below 50 (QS = % completeness – 5 × % contamination). Each genome is assigned a unique MGnify accession, starting with “MGYG” and followed by nine digits. We organise genomes into species clusters at 95% average nucleotide identity using dRep with minimum level of overlap between genomes set to 0.3. If a species cluster includes multiple conspecific genomes, dRep is used to select the highest quality genome as the SR based on completeness, contamination and N50. If a species cluster includes isolate genomes, the highest quality isolate genome is chosen as the SR, regardless of the estimated quality of the MAG(s). GUNC is used to remove potentially chimeric genomes in single genome species clusters, as described previously.

We analyse all genomes that pass quality-filtering using Prokka with default parameters to identify coding sequences. To produce the biome-specific protein catalogue, resulting protein sequences are clustered at 50%, 90%, 95% and 100% amino acid identity using the “linclust” function of MMseqs2^46^ with parameters “--kmer-per-seq 80” (number of k-mers selected per sequence) and “--cov-mode 1 -c 0.8” (minimum coverage threshold of 80%). Protein sequences from both the SRs and the protein catalogue clustered at 90% amino acid identity are further annotated with InterProScan^47^ and eggNOG-mapper.^48^ We identify non-coding RNAs using Infernal v1.1.4, tRNAscan-SE 2.0.9^49^ and Rfam v13.0 covariance models. For species clusters containing multiple conspecific genomes, we compute pan-genomes using Panaroo,^50^ generate a list of core genes (defined as genes present in at least 90% of the conspecific genomes), and generate a species tree in the Newick format for each cluster using genome distances calculated by Mash.

We determine the taxonomy of the SRs using GTDB-Tk, and generate a Kraken 2 database for each catalogue and a Bracken database with k-mer lengths of 50, 100, 150, 200, 250. We generate a Mash sketch for each catalogue that includes all species and strains. Finally, a phylogenetic tree of SRs is generated using IQ-TREE 2 and the multiple sequence alignment from GTDB-Tk. Catalogues generated using pipeline v1.3.1 include additional datasets and annotations. Specifically, we produce a gene catalogue containing the nucleotide sequence of each protein cluster representative from the protein catalogue clustered at 100% amino acid identity. Furthermore, we annotate biosynthetic gene clusters in the SRs using SanntiS^51^ as well as perform viral sequence identification and annotation using VIRify.^52^

### Updating a genome catalogue

To add new genomes to an existing catalogue, we first apply a quality filter to the new genomes using CheckM and remove any genome with QS below 50. We then remove redundancy at the strain level among the new genomes using dRep at 99.9% average nucleotide identity and 0.6 alignment fraction. To identify whether each new genome represents a new species, a new strain of an existing species or an existing strain, we compute the distance between each new genome and its closest genome in the catalogue using Mash. If the distance is < 0.001, the genome is discarded. If the distance is between 0.001 and 0.05, the genome is added to the existing species cluster as a new strain, and if it is > 0.05, the genome is checked for chimerism using GUNC and added to the catalogue as a new species.

For each modified cluster, we determine whether the SR status should be reassigned to one of the newly added genomes. If an isolate is added to a cluster that previously contained exclusively MAGs, the isolate becomes the new SR. If an isolate is added to a cluster that already contains isolates, or a MAG is added to an all-MAG cluster, the new genome becomes the SR if its QS score is at least 110% of the QS score of the existing SR. Finally, the coding sequences in the newly added genomes are annotated using Prokka, complete functional annotation is performed if the new genome becomes a SR, and any affected pan-genomes, and all databases are recalculated to include the new genomes.

### Implementation of the search functionality

Two algorithms are implemented on the MGnify website and API enabling users to compare their own sequences against the catalogues. To search a short sequence (50–5,000 bp) against the SRs of a catalogue or multiple catalogues, we build a k-mer index for SR genome FASTA files using COBS. The search is performed by comparing k-mers in the query sequence against the index of each target catalogue.

To enable users to search genome(s) (MAG and/or isolate genomes) against one or several catalogues, we compute hash-sketches from the SR sequences using sourmash. Users can query the catalogues by submitting their own sourmash-generated sketches, which can be generated programmatically, using the sourmash package and uploaded via the MGnify API. Alternatively, queries via the website accept a genome sequence in FASTA format, which are sketched by a web component in their browser for submission to the server.

### Assessing genome novelty and building a phylogenetic tree

To generate the data shown in Figure 1, we used GTDB release 207 and GTDB-Tk v.2.1.0 to identify the genomes within MGnify catalogues which cannot be classified to family, genus or species level and therefore come from taxa not known in GTDB.

To generate the data shown in Figure 3, we downloaded all genomes from GTDB r207 and filtered them using the associated metadata table as follows. Isolate genomes were identified by the value “none” in the “ncbi_genome_category” field and representatives by filtering out genomes with the value “f” in the “gtdb_representative” field. Completeness, contamination and N50 were also obtained from the metadata table. The GTDB r207 taxonomy for the combined dataset (comprising filtered GTDB isolate genomes, MGnify catalogue MAG SRs, and MGnify catalogue isolate SRs) was used to identify genomes belonging to the same taxonomic class. Classes containing only GTDB isolate genomes were removed. The highest quality genome in each class was determined using the following formula: % completeness – 5 × % contamination + 0.5 × log(N50). A multiple-sequence alignment of the taxonomic marker set for the class representative genomes was produced using the GTDB-Tk v.2.1.0 “identify” and “align” functions with default parameters and the phylogenetic tree built using IQ-TREE v2.1.3 and the LG + R10 model. To generate the MAG and isolate counts for each class, we considered a species an isolate if it was represented by an isolate genome in GTDB or MGnify regardless of any MAGs also present. We counted a species as a MAG if none of its SRs in any of the catalogues were isolates. Each species was therefore only counted once including in cases where the same species was present in more than one catalogue.

## Funding

European Union's Horizon 2020 Research and Innovation programme [862923]; Biotechnology and Biological Sciences Research Council [BB/W002965/1, BB/R015228/1 and BB/V01868X/1]; European Molecular Biology Laboratory core funds; A.A. is supported by a Career Development Award by the Medical Research Council (MR/W016184/1). Funding for open access charge: UK Research and Innovation (UKRI).

## CRediT authorship contribution statement

**Tatiana A. Gurbich:** Conceptualization, Methodology, Software, Writing – original draft, Writing – review & editing, Visualization. **Alexandre Almeida:** Conceptualization, Methodology, Software, Writing – review & editing, Supervision. **Martin Beracochea:** Software. **Tony Burdett:** Software, Data curation. **Josephine Burgin:** Software, Data curation. **Guy Cochrane:** Software, Data curation. **Shriya Raj:** Funding acquisition, Writing – review & editing. **Lorna Richardson:** Conceptualization, Methodology, Writing – original draft, Writing – review & editing, Supervision. **Alexander B. Rogers:** Software, Writing – original draft, Writing – review & editing, Visualization. **Ekaterina Sakharova:** Software. **Gustavo A. Salazar:** Software. **Robert D. Finn:** Conceptualization, Funding acquisition, Methodology, Writing – original draft, Writing – review & editing, Supervision.
