# Supplementary material for: MGnify Genomes: A Resource for Biome-specific Microbial Genome Catalogues
Source: J Mol Biol. 2023 Jul 15;435(14):168016. doi: 10.1016/j.jmb.2023.168016 (PMC10318097; doi:10.1016/j.jmb.2023.168016)
Supplement: Supplementary Data 1 [file mmc1.pdf]

Class name	Total number of species in class	Number of species with a cultured representative in GTDB or MGnify catalogues	Number of species with only MAGs as species representatives
ABY1	2	0	2
Acidimicrobiia	73	17	56
Acidobacteriae	79	77	2
Actinomycetia	4806	4730	76
Alphaproteobacteria	4056	3661	395
Anaerolineae	31	23	8
Aquicultoria	2	1	1
Bacilli	3668	3065	603
Bacteriovoracia	14	9	5
Bacteroidia	4167	2573	1594
Bdellovibrionia	14	12	2
Bdellovibrionia_A	1	0	1
Bipolaricaulia	5	1	4
BMS3Abin14	2	0	2
BMS3BBIN04	1	0	1
Brachyspirae	18	15	3
Bradymonadia	12	7	5
Brevinematia	4	1	3
Brocadiae	8	6	2
Caldisericia	6	5	1
Campylobacteria	318	294	24
Chlamydiia	48	43	5
Clostridia	4337	1342	2995
Coriobacteriia	798	179	619
Cyanobacteriia	524	503	21
Deferribacteres	17	9	8
Dehalobacteriia	8	3	5
Dehalococcoidia	73	9	64
Deinococci	110	107	3

Desulfitobacteriia	33	33	0
Desulfobacteria	38	33	5
Desulfobulbia	24	23	1
Desulfotomaculia	38	36	2
Desulfovibrionia	141	111	30
Desulfurellia	8	6	2
Desulfurobacteriia	9	8	1
DTU015	3	1	2
Elusimicrobia	39	2	37
Endomicrobia	5	2	3
Fibrobacteria	45	19	26
Fimbriimonadia	4	3	1
Fusobacteriia	93	69	24
Gammaproteobacteria	6469	5948	521
Gemmatimonadetes	18	6	12
Gracilibacteria	3	0	3
Halanaerobiia	26	25	1
Hydrothermia	1	0	1
Ignavibacteria	5	2	3
JAAXHH01	1	0	1
JAEDAM01	9	2	7
JdFR-76	2	0	2
Kapabacteria	1	0	1
Kiritimatiellae	83	5	78
Koll11	4	1	3
Lentisphaeria	57	5	52
Leptospirae	75	73	2
Marinisomatia	49	7	42
MSB-5A5	2	1	1
Negativicutes	297	135	162
Nitrospinia	6	1	5
Nitrospiria	13	11	2

NPL-UPA2	1	0	1
Oligoflexia	9	8	1
Ozemobacteria	2	0	2
Paceibacteria	24	0	24
Peptococcia	13	3	10
Phycisphaerae	28	14	14
Planctomycetia	160	124	36
Polyangia	23	21	2
Rhodothermia	46	34	12
RUG730	1	0	1
Saccharimonadia	92	31	61
SAR324	11	0	11
SHA-98	1	0	1
SM23-61	1	0	1
Spirochaetia	193	94	99
Synergistia	29	25	4
SZUA-365	2	0	2
SZUA-79	1	0	1
Thermoanaerobacteria	30	29	1
Thermodesulfovibrionia	17	9	8
Thermoleophilia	23	21	2
Thermosediminibacteria	10	9	1
Thermotogae	52	51	1
UBA1135	5	3	2
UBA1144	8	0	8
UBA1177	5	0	5
UBA11872	2	0	2
UBA2361	1	0	1
UBA2968	4	0	4
UBA3569	2	0	2
UBA4151	1	0	1
UBA6624	1	0	1

UBA6919	1	0	1
UBA727	1	0	1
UBA7883	2	0	2
UBA796	5	0	5
UBA8108	4	0	4
UBA8248	2	0	2
UBA890	1	0	1
UBA9160	3	1	2
UBA9942	2	0	2
Vampirovibrionia	153	4	149
Verrucomicrobiae	188	92	96
Vicinamibacteria	14	2	12
WGA-4E	2	0	2
Xenobia	7	0	7
Zetaproteobacteria	11	9	2
